# The outcome of surgical management for giant retinal tear more than 180°

**DOI:** 10.1186/1471-2415-14-86

**Published:** 2014-06-27

**Authors:** Sherif A Dabour

**Affiliations:** 1 Department of Ophthalmology, Zagazig University, Zagazig, Egypt

**Keywords:** Giant retinal tear, Pars plana vitrectomy, Rhegmatogenous retinal detachment, Scleral buckling, Retinal slippage

## Abstract

**Background:**

To evaluate the surgical outcome for management of giant retinal tear (GRT) more than 180° by combined pars plana vitrectomy (PPV), encircling scleral buckle, 360° Laser endophotocoagulation, and silicon oil tamponade.

**Methods:**

This was a Prospective, interventional case series study. Twenty four eyes of 23 patients suffering from GRT more than 180° underwent PPV combined with encircling scleral buckle and 360° laser retinopexy of the peripheral retina followed by silicon oil tamponade. All patients were followed up for at least six months.

**Results:**

Complete anatomical success (retinal attachment after silicone oil removal) was achieved in 20 (83.3%) eyes at the end of follow-up, while incomplete anatomical success (retinal redetachment under or after removal of silicon oil) occurred in four (16.7%) eyes. At the end of follow-up period (mean of 13.7 months + 6.5), improvement of best-corrected visual acuity was achieved in 22 (91.7%) eyes. Preoperative best-corrected visual acuity ranged from HM to 0.15 while postoperative visual acuity ranged from HM to 0.9. Retinal slippage did not occur in any case. Additionally, removal of the clear crystalline lens in phakic eyes was not necessary in the primary intervention.

**Conclusions:**

GRT more than 180° can be effectively treated with PPV coupled with encircling scleral buckle, 360° laser retinopexy and silicon oil tamponade with no incidence of retinal slippage. In this complex procedure, concurrent encircling buckle the primary intervention may contribute to high chance of success.

## Background

Giant retinal tear (GRT) is a full-thickness retinal break extending circumferentially for ≥90° (≥3 clock hours) of the retina. It is a major and challenging ophthalmological problem with a wide variety of surgical techniques for unfolding and sealing the retinal tear [1-11].

Despite improvement in the surgical maneuvers and tamponade agents, recurrence of the detachment still occurs due to several factors, such as reopening of the tear, formation of a new tear, or extension of the existing tear due to concurrent proliferative vitreoretinopathy (PVR). Furthermore, the surgical procedure is more challenging to reattach the retina when the GRT circumference is more than 180° as there is a high risk of failure and retinal slippage [4,8,9,12].

There is a debate about the role of scleral buckle procedure combined with PPV in the surgical management of GRT. While some surgeons consider it a beneficial procedure to help attachment of the retina because it minimizes vitroretinal tractional forces, others consider it a real cause of posterior retinal slippage due to its role in changing ocular contour and scleral shortening relative to retina. Alternatively, some surgeons consider it only in recurrent cases [2,4-8,13,14].

In the study herein, the author studied the safety and efficacy of combined PPV, encircling scleral buckle, 360° laser retinopexy and postoperative silicon oil tamponade in management of GRT more than 180°.

## Methods

Twenty four eyes of 23 consecutive patients having GRT more than 180° were included in this prospective study. Informed written consent was obtained from each patient to do pars plana vitrectomy and scleal buckle for treatment of giant retinal tear retinal. All surgeries were done in Zagazig University Hospital between June 2005 and May 2013. The study was approved by the institutional review board of Zagazig University (ZUIRDB) at the start of the study and adhered to the tenets of Helsinki Declaration.

Three patients (three eyes) had a history of blunt ocular trauma, six patients (seven eyes) had a history of cataract extraction prior to GRT, while one patient had a history of LASIK. Two patients had Marfan’s syndrome. Eyes with a history of previous vitreous surgery were excluded from the study.

### Surgical technique

All eyes were operated under general anesthesia. At the beginning surgery, 360° periotomy was done followed by slinging of the four recti muscle and placement of an encircling equatorial band No. 41 (3.5 mm), but not tied till the retinal is flat under the PFCL.

Conventional 3-port pars plana vitrectomy (PPV) procedure using 20-gauge vitrectomy system coupled with non-contact wide field viewing system (BIOM, OCULUS Optikgeräte GmbH, Wetzlar, Germany). Triamcinolone assisted vitrectomy was performed and then perfluorocarbon liquid (PFCL) was injected into the vitreous cavity to unroll the retina and displace the subretinal fluid. This was followed by diathermy of the edges of the tear, excision of the anterior flap, and smoothening of the edges of the posterior flap. Meticulous removal of the peripheral vitreous base under wide field viewing with indentation with all efforts made to remove as much vitreous as possible. Under PFCL tamponade, 360° laser (several rows extended up to the retinal periphery) was applied to seal the retina and cryopexy to the two horns of GRT, if there was any radial extension. Finally, tightening the encircling silicon 360° band followed by direct PFCL/silicon oil (5000 Cs) exchange. The height of the buckle aimed to be relatively low and broad to minimize radial folds formation. All patients were instructed for postoperative face down for 20 hours daily for at least seven days. All surgeries were done by one surgeon (SAD). In phakic eyes, the lens was spared in all cases. This treatment was a part of standard patient care and not specific for the study.

Silicon oil removal, with or without cataract surgery, was planned within 4–6 months from the initial surgery. All patients were followed up regularly for at least six months after silicone oil removal with complete ophthalmological examination each visit with special attention to best-corrected visual acuity (BCVA), lens status, IOP and peripheral retinal state.

Complete anatomical success was defined as complete retinal attachment after silicone oil removal at the 6th postoperative month, while incomplete success was considered in eyes were the retina remained detached under silicon oil or redetached after silicon oil removal.

## Results

Twenty four eyes (23 patients, 19 males and four females) suffering from GRT more than 180° were included in the current study. The mean age was 37.1 ± 10.6 years (range: 15–52 years). PVR was grade A in 13 eyes, grade B in six eyes, and grade C in five eyes. At the initial procedure, 18 eyes were phakic, five eyes were pseudophakic and one eye was aphakic. In all phakic eyes, the lens was spared in the primary intervention. Table 1 shows the clinical characteristics and demographics of the study subjects. The mean follow-up period was 13.7 ± 6.5 (range: 6–26 months).

**Table 1:** Clinical characters of the eyes with giant retinal tear more than 180°

**Case no.**	**Age (years)**	**Sex**	**Eye**	**Extent of GRT**	**Macula status**	**PVR**	**Lens**	**Anatomical success**	**Pre-operative BCVA**	**BCVA at the end of Follow-up**	**Follow-up period (months)**
1	40-50	Male	OS	240°	Off	A	Phakic	Complete	HM	0.3	13
2	50-60	Female	OD	210°	Off	B	Phakic	Complete	CF	0.5	7
3	40-50	Male	OS	190°	On	A	Phakic	Complete	0.15	0.9	15
4	-40-50	Male	OS	210°	Off	CP3	Phakic	Complete	HM	0.3	22
5	30-40	Male	OD	240°	Off	A	Phakic	Complete	CF	0.1	11
6	50-60	Male	OD	250°	Off	A	Phakic	Complete	CF	0.3	20
7	15-20	Male	OS	210°	Off	B	Phakic	Complete	CF	0.3	24
8	40-50	Male	OD	240°	Off	B	Phakic	Complete	HM	0.3	20
9	30-40	Male	OS	270°	On	A	Pseudophakic	Complete	0.05	0.7	20
10	20-30	Female	OS	210°	Off	A	Phakic	Complete	HM	0.2	9
11	30-40	Male	OD	210°	Off	A	Phakic	Complete	CF	0.4	7
12	40-50	Male	OD	240°	Off	CP6	Phakic	Complete	CF	0.1	15
13	30-40	Male	OD	240°	Off	A	Aphakic	Incomplete	CF	CF	26
14	30-40	Male	OS	300°	Off	A	Phakic	Complete	HM	0.2	6
15	20-30	Female	OS	250°	Off	B	Phakic	Complete	CF	0.3	10
16	30-40	Male	OD	330°	Off	A	Pseudophakic	Incomplete	HM	HM	7
17	40-50	Male	OD	190°	Off	B	Pseudophakic	Complete	CF	0.2	24
18	50-60	Female	OS	240°	Off	B	Phakic	Complete	CF	0.1	15
19	30-40	Male	OD	240°	Off	A	Phakic	Complete	HM	0.05	18
20	30-40	Male	OD	210°	Off	A	Pseudophakic	Complete	HM	0.1	8
21	15-20	Male	OS	240°	Off	CP3	Phakic	Complete	CF	0.1	9
22	50-60	Male	OS	240°	Off	CP6	Pseudophakic	Incomplete	HM	0.01	7
23	30-40	Male	OS	200°	Off	CP3	Phakic	Incomplete	HM	0.05	9
24	30-40	Male	OS	240°	On	A	Phakic	Complete	0.15	0.8	7

*Abbreviations: PVR*, proliferative vitreoretinopathy; *BCVA*, best corrected visual acuity; *HM*, hand motion; *CF*, counting fingers.

The mean circumference of the GRT was 235.0° ± 32.4° (range: 190–330°). The macula was attached in three (12.5%) eyes while detached in the remaining 21 (87.5%) eyes. Preoperative best-corrected visual acuity (BCVA) ranged from hand motion (HM) to counting fingers (CF) in macula-off patients, while decimal BCVA in eyes with attached macula was 0.15 in two eyes and 0.05 in one eye.

Anatomical attachment with primary procedure was achieved in 20 (83.3%) eyes. In the remaining four (16.7%) eyes, retinal detachment recurred in two eyes during silicon oil tamponade due to PVR and in the other two eyes redetachment occurred after silicone oil removal. These eyes were managed by reinjection of silicone oil and further laser retinopexy to the whole peripheral retina while the buckle in place. In three eyes, the retina was successfully attached in the second procedure with no recurrence after the removal of silicon oil while in the remaining eye, the visual acuity was poor and the patient refused further surgery.

At the end of follow-up period, BCVA improved in 21 (87.5%) eyes, did not change in two (8.3%) eyes, and worsened in one (4.2%) eye. Fourteen eyes, out of the 18 phakic eyes, developed cataract during the follow-up period and were managed by phacoemulsifaction and lens implantation during silicone oil removal. Subretinal hemorrhage occurred in one eye during application of laser; fortunately, it was small amount and nasal and managed by observation and follow-up. Two patients developed secondary glaucoma after silicon oil removal and were managed by sub-scleral trabeculectomy with adjuvant Mitomycin C.

## Discussion

In the study herein, GRT more than 180° were successfully treated with combined PPV and encircling scleral buckle, and the tear was further stabilized with adjuvant 360° laser retinopexy and postoperative oil tamponade. This approach afforded good anatomical and visual outcomes.

Management of GRT is a challenging surgical problem with many different approaches to manage; however, when the GRT is more than 180°, the intervention is rather complex.

Adjuvant scleral buckling in the management of GRT is still debatable. In this study, an encircling buckle was routinely placed in all cases in the primary intervention irrespective to grade of PVR. The rationale is that GRT more than 180° has a high risk of recurrence and it is essential to target successful attachment in the primary surgery as reoperation is rather complex in these cases and the outcome is often poor. Also, previous studies reported that the larger the size of the giant tear, the more the risk of redetachment [8]. Additionally, GRT more than 180° often involve the inferior retina and thus buckle is often needed, in addition to silicon oil tamponade, to support the inferior quadrant.

Some surgeons prefer PPV without buckling in management of GRT, provided that traction is relieved after thorough vitrectomy. Additionally, buckling can complicate the closure of GRT by causing a gaping of retinal tissue, redundant retinal folds when the buckle is tightened, fish-mouthing and increased tendency of posterior retinal slippage [3,4,6,14].

Conversely, other surgeons prefer adjunctive buckling in as a primary procedure aiming to reduce the failure rate. Their rationale was that scleral buckling reduces the early and late tractional forces, and supports areas of undetected retinal breaks [15,16]. Meanwhile, other surgeons reserve scleral buckling only for second intervention [2].

Previous studies reported successful repair of GRT with PPV and 360° laser retinopexy without scleral buckling. Kreiger et al. [6] previously treated 11 cases with GRT with PPV and silicon oil tamponade with strong emphasis on extended laser treatment to the whole peripheral retina to create strong adhesion and to minimize secondary tears due to anterior PVR. They didn’t place a scleral buckle in their study. Their procedure was successful in 10 (90.9%) eyes and they experienced recurrence in only one (9.1%) case occurred as the result of posterior PVR.

Similarly, Ambresin et al. [3] treated a series of 18 eyes with the same technique and they experienced successful retinal attachment in 16 (88.8%) eyes and recurrence in only two eyes.

On the other hand, in a prospective randomized comparative study conducted by Sharma et al. [16], they used 360° degree 9 mm silicone band buckle in 10 cases and none in 11 cases. They reported that the primary success was 100% in sclera buckle group as compared to 37.5% in non sclera buckle group, and that resurgeries were required in 8 out of11 cases in non-scleral buckle group. The final visual acuity was better in eyes treated with scleral buckle.

Also, the intraoperative use of PFCL was essential to unfold the retina, to displace subretinal fluid and blood, and to stabilize the retina-providing counter attraction for any membrane dissection, and to avoid the need for drainage retinotomy [4,8]. Retinal slippage in the presence of buckle was avoided by doing direct PFCL/silicon exchange and tightening of the scleral buckle was done before exchange to ensure complete silicon fill. It is still difficult to compare these results with previous reports due to the difference in the clinical characteristics of the study subjects and surgical procedure.

Controversy remains whether lens extraction is necessary or not in the management of fresh giant tears. The advantage of lens removal is the better visualization of vitreous base. In this series, it was found that lens removal was not necessary and may minimize the surgical trauma in such complex procedure. In addition, intraocular lens power calculation is often inaccurate in eyes with GRT when the macula is off. Moreover, the use of wide angle viewing systems coupled with indentation for giant tear surgery improves the ability to see the peripheral retina in phakic and pseudophakic eyes and makes thorough vitreous base shaving feasible.

This doesn’t agree with Kreiger et al. [6], who believed that a lensectomy is necessary for optimal removal of the basal vitreous and provides excellent visualization postoperatively for photocoagulation and avoids subsequent cataract surgery. Sharma et al. [16] considered lens removal only in cataractous eyes, subluxated lenses or the presence of PVR are the main indications for lens removal in GRT.

Also, many eyes with GRTs are often highly myopic and the pars plana region is often wide and broad. This anatomic variation allows adequate exposure of the vitreous base with less risk of lens touch. However, the initial surgery for GRT should not be compromised to preserve the lens. In the current study, cataract developed in 14 of the 18 phakic eyes and phacoemulsifaction was done during silicon oil removal thus avoiding disadvantages of multiple surgeries.

## Conclusions

Our results suggest that that PPV combined with scleral buckle, 360° laser photocoagulation and postoperative oil tamponade is effective in the management of GRT more than 180°. This approach afforded good anatomical and visual outcomes.

## Competing interest

The author has no competing interest.

## Pre-publication history

The pre-publication history for this paper can be accessed here:

http://www.biomedcentral.com/1471-2415/14/86/prepub
